# Juvenile polyposis syndrome affecting the stomach: A case report

**DOI:** 10.1186/1752-1947-2-314

**Published:** 2008-09-30

**Authors:** Steven Kelly, Simon Dwerryhouse, Peter Safranek, Richard Hardwick

**Affiliations:** 1 Cambridge Oesophagogastric surgery centre, Addenbrookes hospital, Cambridge, UK

## Abstract

**Introduction:**

Juvenile polyposis syndrome(JPS) is a rare autosomal dominant inherited condition. Hamartomatous polyps can affect the entire gastrointestinal tract but usually predominate in the colon. In this case report we present an unusual case of JPS that presented with massive gastric polyposis requiring a total gastrectomy.

**Case presentation:**

A 51-year-old man presented with symptoms of gastric outlet obstruction and upper gastrointestinal bleeding. Gastroscopy showed massive gastric polyposis with a large antral polyp that had prolapsed through the pylorus causing gastric outlet obstruction. Initially endoscopic polypectomy was performed, but due to progressive symptoms a total gastrectomy was then performed. Histology confirmed massive gastric juvenile polyposis.

**Conclusion:**

Massive gastric polyposis is an uncommon manifestation of juvenile polyposis syndrome. This case illustrates important principles in managing this condition.

## Introduction

Juvenile polyposis syndrome (JPS) is a rare autosomal dominant inherited condition. These juvenile polyps can occur anywhere in the gastrointestinal tract but usually predominate in the colon. We report an unusual case of massive gastric polyposis occurring in a patient with juvenile polyposis syndrome. Due to the severity of the disease he required total gastrectomy.

## Case presentation

A 51-year-old man with known juvenile polyposis syndrome had been in a gastrointestinal polyposis screening program which had starting twenty years earlier when he was initially diagnosed with gastric and colonic juvenile polyps. Two years ago he required a laparotomy and small bowel resection for a large intussuscepting jejunal juvenile polyp causing obstruction.

Clinically there were no extraintestinal manifestations of hamartomatous polyposis syndromes. There was a strong family history of gastrointestinal malignancy. The patient had two brothers both of whom were diagnosed with colon cancer in their fifties. The patient had two children in their twenties, both of whom had colonic juvenile polyps and one had had surgery for colonic cancer.

**Figure 1:**
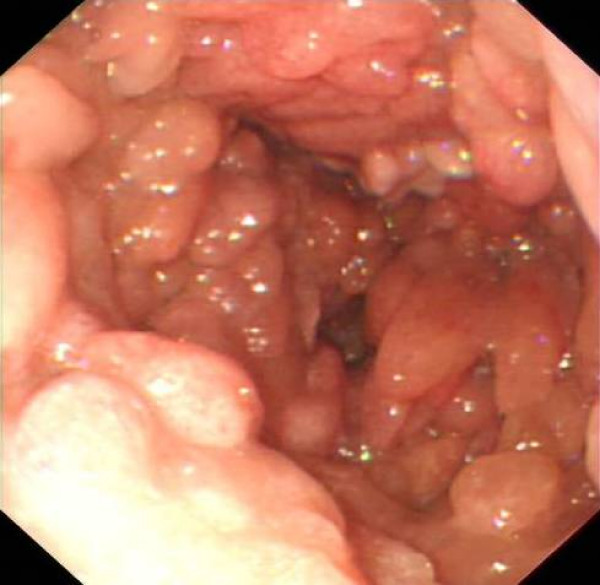
Endoscopic view of massive gastric polyposis.

**Figure 2:**
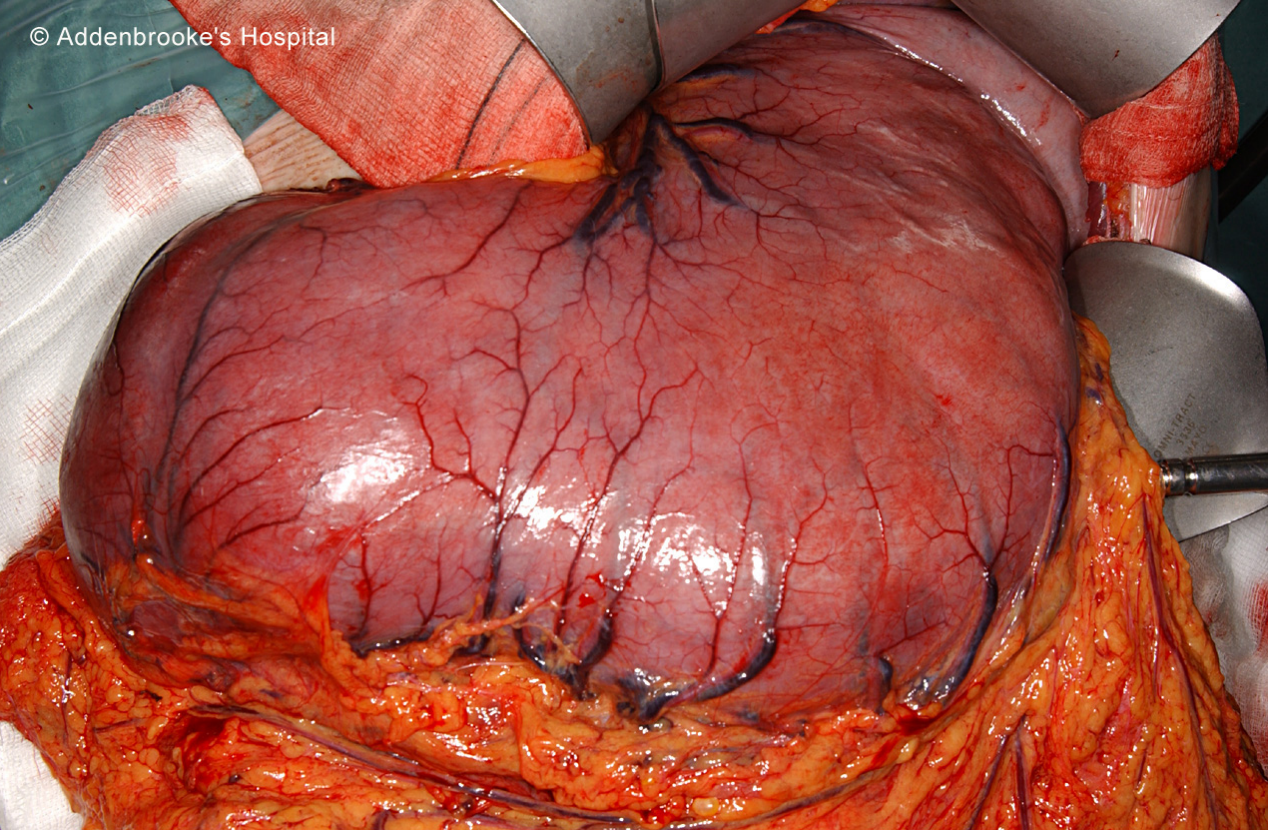
Intra-operative photo of stomach after mobilisation.

The patient presented with symptoms of vomiting, early satiety, weight loss and upper gastrointestinal bleeding. Serum haemoglobin measurement was 5 g/dl. Gastroscopy and barium meal examination revealed massive gastric polyposis with a prominent gastric antral polyp prolapsing through the pylorus and causing pyloric obstruction (figure 1). Attempts were made at endoscopic debulking of the gastric polyps but this had been unsuccessful. Surgery was therefore advised given the worsening symptoms of gastric outlet obstruction and repeated episodes of upper gastrointestinal bleeding. The patient underwent a vagal sparing total gastrectomy with a 50 cm Roux-en-Y jejunal reconstruction stapled end to side to the oesophagus. This was a difficult procedure as the stomach was very large (figure 2). There were no postoperative complications and he was discharged ten days post-surgery.

**Figure 3:**
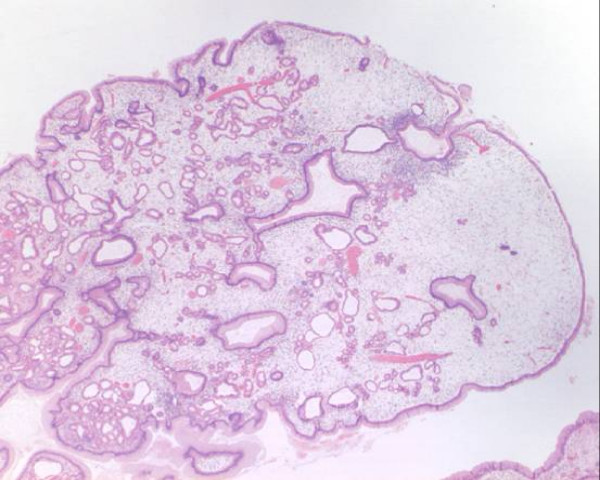
Histology of juvenile polyp showing characteristic features of dense stroma and dilated cystic spaces.

Histology of the surgical specimen showed massive juvenile gastric polyposis (figure 3). There was focal intestinal metaplasia and mild dyplasia. No evidence of malignancy was found.

Genetic mutational analysis of the BMPR1A and SMAD4 genes was not performed.

## Discussion

Juvenile polyposis syndrome is a rare autosomal dominant inherited condition. It is characterised by the presence of juvenile polyps that can occur in the stomach or small and large intestine. Histologically juvenile polyps are hamartomas that have normal epithelium with a dense stroma, an inflammatory infiltrate, and a smooth surface with dilated, mucus-filled cystic glands in the lamina propria.

Juvenile polyposis syndrome can be diagnosed if any one of the following criteria is met (Jass criteria) [1].

• More than five juvenile polyps in the colorectum **OR**

• Multiple juvenile polyps throughout the gastrointestinal tract **OR**

• Any number of juvenile polyps *and *a family history of juvenile polyps

The diagnosis of juvenile polyposis syndrome can be made in this case as there was evidence of multiple juvenile polyps throughout the gastrointestinal tract and there was also a family history of juvenile polyps.

On searching the literature, the prevalence of massive gastric polyposis in patients with juvenile polyposis syndrome has never been reported but we believe it is likely to be low.

Mutations in two different genes (*BMPR1A *and *SMAD4*) are known to cause juvenile polyposis syndrome in 20% of cases each [2]. Genotypic and phenotypic correlations have generally been poor with these gene mutations but a recent report has shown a strong correlation between massive gastric polyposis and the presence of a SMAD4 gene mutation versus the BMPR1A or no mutation [3]. Although the patient in this case did not have genetic analysis performed, we postulate that he may be more likely to have a gene mutation in the SMAD4 gene.

Although most juvenile polyps are benign, malignant transformation can occur. This has been estimated to occur in 9 to 50% of cases with juvenile polyposis syndrome [4]. Most of the malignant transformation occurs as colorectal cancer but can occur as gastric or small bowel cancer.

Individuals with a proven SMAD4/BMPR1A mutation, or a clinical diagnosis of JPS by the Jass criteria, should receive education and screening to reduce the risk of malignancy. Recommended screening involves colonoscopy and gastroscopy starting at 15 years of age and repeated every three years [5]. All polyps should be removed and when the polyp burden becomes large surgical resection should be performed. Approximately 75% of patients with JPS have an affected parent. Therefore 25% of cases are due to de novo mutations. If an individual with JPS is found to have a mutation then the other family members can be offered genetic testing around the age of 15 years. Family members at risk of JPS but with no identified mutation should have colonoscopic screening starting at 15 years of age and repeated every ten years.

## Conclusion

Massive gastric polyposis secondary to juvenile polyposis syndrome is a rare condition. This case report illustrates the importance of making the diagnosis of juvenile polyposis syndrome and entering the patient into a screening program. People with gastric polyposis should be treated initially with endoscopic polypectomy, however a total gastrectomy may sometimes be necessary.

## Competing interests

The authors declare that they have no competing interests.

## Authors' contributions

SK was involved in writing of the case report. RH was involved in the review and re-writing of the case report. All authors were involved in the patient's care.

## Consent

Written consent was obtained from the patient for the publication of the case and the clinical images. A copy of the written consent is available for review by the Editor-in-Chief of this journal.
